# Seismic imaging and petrology explain highly explosive eruptions of Merapi Volcano, Indonesia

**DOI:** 10.1038/s41598-018-31293-w

**Published:** 2018-09-12

**Authors:** S. Widiyantoro, M. Ramdhan, J.-P. Métaxian, P. R. Cummins, C. Martel, S. Erdmann, A. D. Nugraha, A. Budi-Santoso, A. Laurin, A. A. Fahmi

**Affiliations:** 1 Global Geophysics Research Group, Faculty of Mining and Petroleum Engineering, Bandung Institute of Technology, Bandung, 40132 Indonesia; 2 Research Center for Disaster Mitigation, Bandung Institute of Technology, Bandung, 40132 Indonesia; 3 Study Program of Earth Sciences, Faculty of Earth Sciences and Technology, Bandung Institute of Technology, Bandung, 40132 Indonesia; 4 Agency for Meteorology, Climatology and Geophysics, Jakarta, 10720 Indonesia; 5 ISTerre, IRD R219, CNRS, Université de Savoie Mont Blanc, Le Bourget-du-Lac, France; 6 Institut de Physique du Globe de Paris, Université Sorbonne-Paris-Cité, CNRS, Paris, France; 7 Research School of Earth Sciences, Australian National University, Canberra, ACT 2601 Australia; 8 Institut des Sciences de la Terre d’Orléans (ISTO), Université d’Orléans-CNRS-BRGM, Orléans, France; 9 Center for Volcanology and Geological Hazard Mitigation, Geological Agency, Bandung, 40122 Indonesia

## Abstract

Our seismic tomographic images characterize, for the first time, spatial and volumetric details of the subvertical magma plumbing system of Merapi Volcano. We present P- and S-wave arrival time data, which were collected in a dense seismic network, known as DOMERAPI, installed around the volcano for 18 months. The P- and S-wave arrival time data with similar path coverage reveal a high Vp/Vs structure extending from a depth of ≥20 km below mean sea level (MSL) up to the summit of the volcano. Combined with results of petrological studies, our seismic tomography data allow us to propose: (1) the existence of a shallow zone of intense fluid percolation, directly below the summit of the volcano; (2) a main, pre-eruptive magma reservoir at ≥ 10 to 20 km below MSL that is orders of magnitude larger than erupted magma volumes; (3) a deep magma reservoir at MOHO depth which supplies the main reservoir; and (4) an extensive, subvertical fluid-magma-transfer zone from the mantle to the surface. Such high-resolution spatial constraints on the volcano plumbing system as shown are an important advance in our ability to forecast and to mitigate the hazard potential of Merapi’s future eruptions.

## Introduction

**Figure 1:**
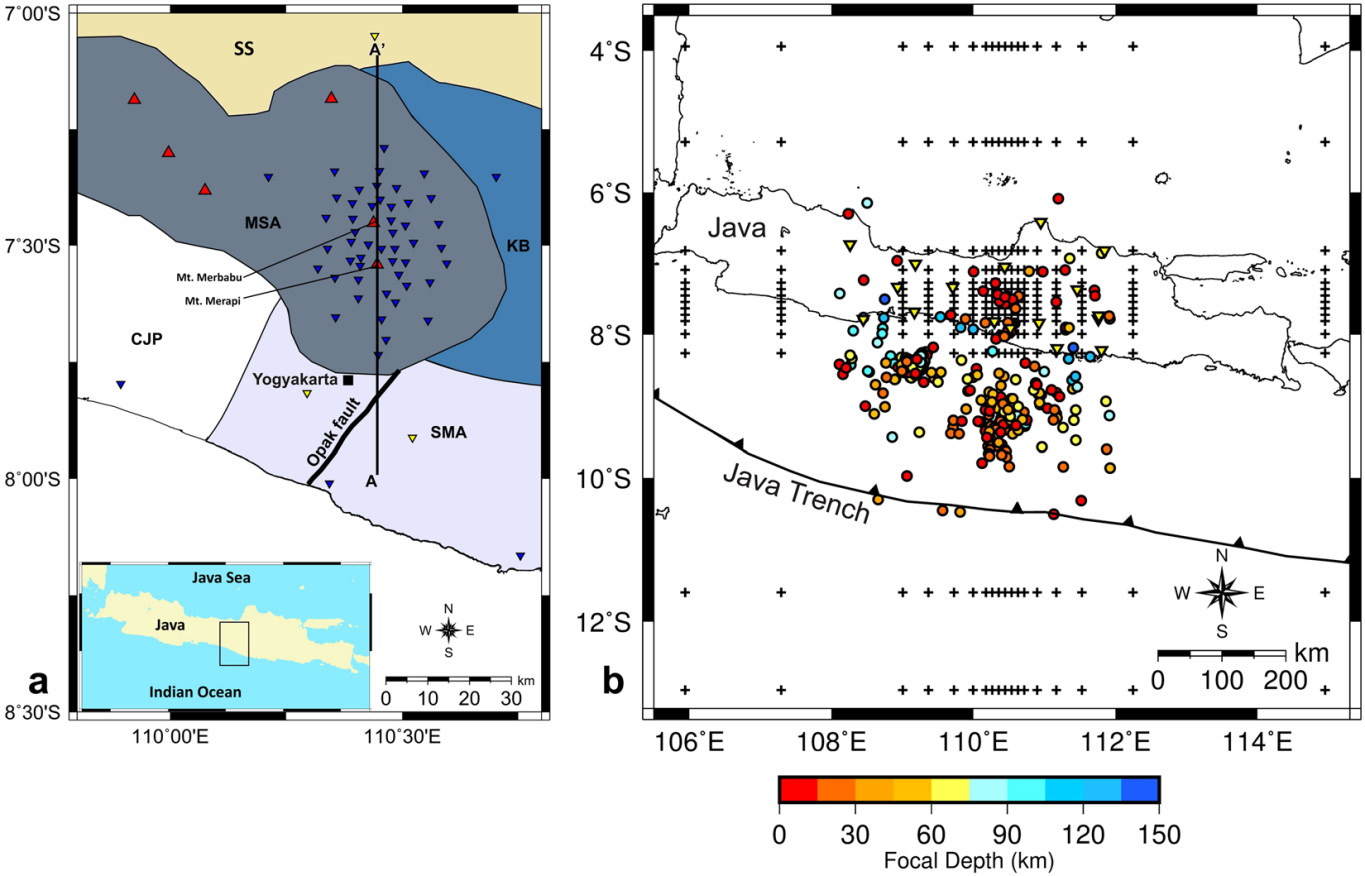
Maps of the study area. (**a**) The main structural units of central Java: Central Java Province (CJP), Modern Sunda Arc (MSA), Sunda Shelf (SS), Kendeng Basin (KB), and Southern Mountain Arc (SMA) (modified from^1^). Symbols: blue and yellow reverse triangles depict the distribution of seismographic stations of the DOMERAPI and BMKG networks, respectively, and red triangles represent volcanoes. The South-North (A-A’) line shows the location of vertical sections presented in Figs 3 and 4. (**b**) Epicentral distribution of relocated events upon SIMULPS12 using a 3-D velocity structure and grid nodes used for tomographic inversions (crosses). Yellow reverse triangles depict the locations of the BMKG stations. Figures 1–3 were produced using the Generic Mapping Tools (GMT) by Wessel and Smith^34^.

Mt. Merapi is Indonesia’s most frequently erupting volcano, which forms part of the Modern Sunda Arc (MSA)^1,2^. Merapi experiences Volcanic Explosivity Index (VEI) 1–2 eruptions roughly once every 6 years, a VEI 3 eruption once every few decades, and a VEI 4 eruption once in a century^3^. These eruptions pose a major threat to Yogyakarta, a cultural and university center with a total population of more than 3.5 million people located on the southern flank of the volcano and close to the active Opak Fault (Fig. 1a).

Because of its frequent activity and high potential for destruction and fatalities, Merapi has been the focus of many studies by researchers worldwide. Many scientists were alarmed by the change in Merapi’s behavior from over two decades of VEI-1 and VEI-2 eruptions to the VEI-4 eruption in 2010^4^. Since the beginning of the 20^th^ century Merapi has experienced 22 eruptions, most of them involving lava dome production and collapse, resulting in pyroclastic flows. The 2010 eruption, in contrast, involved lava dome production at the extraordinary rate of 25 m^3^s^−1^, a hundred times that of previous eruptions, as well as correspondingly larger gas emissions, seismic energy release, eruption plume height, and volume of erupted lava^4^. The distinctive character of the seismicity, gas emissions, and lava petrology of the 2010 eruption all suggest that the difference with respect to previous post-19^th^ century eruptions was the unusually rapid ascent of a large volume of volatile-rich magma sourced from depths >8 km^4–7^.

A large number of petrological studies have already proposed models for Merapi’s magma plumbing system, ranging from those that suggest the presence of many small magma reservoirs throughout the crust^8–11^ to those that favor storage in one or more main zones^5,6,12^. Apart from quantitative differences and large uncertainties of the estimates for the depth distribution of the magma storage zones (for a summary see Supplementary Information and our later discussion), it is important to highlight that petrological estimates cannot unequivocally identify main storage zones or indeed constrain the full spatial or volumetric extent of magma storage zones in the crust. They can only identify depth ranges from which magma or magmatic cumulates have erupted.

Previous geophysical studies have either focused on the shallow system below Merapi at depths of <10 km^13–17^, or relied on low-resolution (~15 km) seismic arrival time and ambient noise tomographic imaging to identify potential magma reservoirs^18–20^. This imaging revealed a strong and extensive low-velocity anomaly about 25 km NE of Merapi that extends from the surface to the mid-crust, and merges into a deeper anomaly inclined southwards towards the subducting slab. It is possible to interpret the mid-crustal part of this anomaly as a magma reservoir consisting of a solid matrix with pockets of partial melt^19^, but such a complicated reservoir involving considerable lateral transport begs the question of how large volumes of volatile-rich magma can be rapidly delivered to the surface to sustain the type of explosive eruption that occurred in 2010. Clearly, accurately imaging Merapi’s magma plumbing system throughout the crust is critical for forecasting and mitigating the hazard potential of future eruptions.

## New, high-resolution seismic tomograms

DOMERAPI, a French-Indonesian collaborative project, deployed a seismograph network of 46 broad-band seismometers in the period from October 2013 to mid-April 2015, with an inter-station distance of ~4 km providing by far the densest coverage of seismographic stations ever used on Merapi (Fig. 1a). The DOMERAPI data were combined with data of the permanent seismographic network of the Indonesian Agency for Meteorology, Climatology and Geophysics (BMKG) to provide better constraints on hypocenter estimates by extending spatial coverage of the data. This was crucial in achieving high precision hypocenter determinations^21^, since the DOMERAPI stations were placed around Mt. Merapi, while most seismic events occurred along the Java trench to the south of the study region (Fig. 1b). All seismic events were relocated using a double-difference earthquake location algorithm^22^. The jointly processed DOMERAPI and BMGK data produced a new, high-quality catalog^21^ comprising 358 events used to undertake the high-resolution tomographic imaging of Merapi presented here.

We have performed joint inversion of the arrival time data to image the Vp and Vp/Vs structure below Merapi in exceptional detail, from below the volcano’s summit to a depth of ~20 km below MSL. We have used the program SIMULPS12^23^, which applies an iterative, damped least squares algorithm to simultaneously calculate the 3-D Vp and Vp/Vs structures and hypocentral adjustments. The Vp/Vs structure was inverted for using S-P times instead of separate estimates of Vs and Vp, which is considered a more robust approach^24^ given that the timing errors for S waves are usually larger than those for P waves.

For our joint inversion of P-wave velocity and Vp/Vs ratios, we have used comparable ray coverage for P and S waves with 5042 phases each, to minimize the possibility that dissimilarities in resulting images are caused by effects of regularization related to differences in data sampling^23^. Figure 1b shows the grid nodes employed in the inversions, i.e. 10 km by 10 km around Merapi, while the vertical grid spacing is 5 km down to 30 km depth and coarser for deeper parts. For an initial reference velocity model we have used a 1D Vp model for central Java^18^ with Vp values ranging from 4.3 km/sec at a depth of 3 km to 8.3 km/sec at a depth of 210 km (see Supplementary Fig. 1). The associated 1D Vs model was derived using a Vp/Vs ratio of 1.73 obtained from the Wadati diagram constructed using the combined DOMERAPI and BMKG data sets^21^.

## Merapi’s magma plumbing system

**Figure 2:**
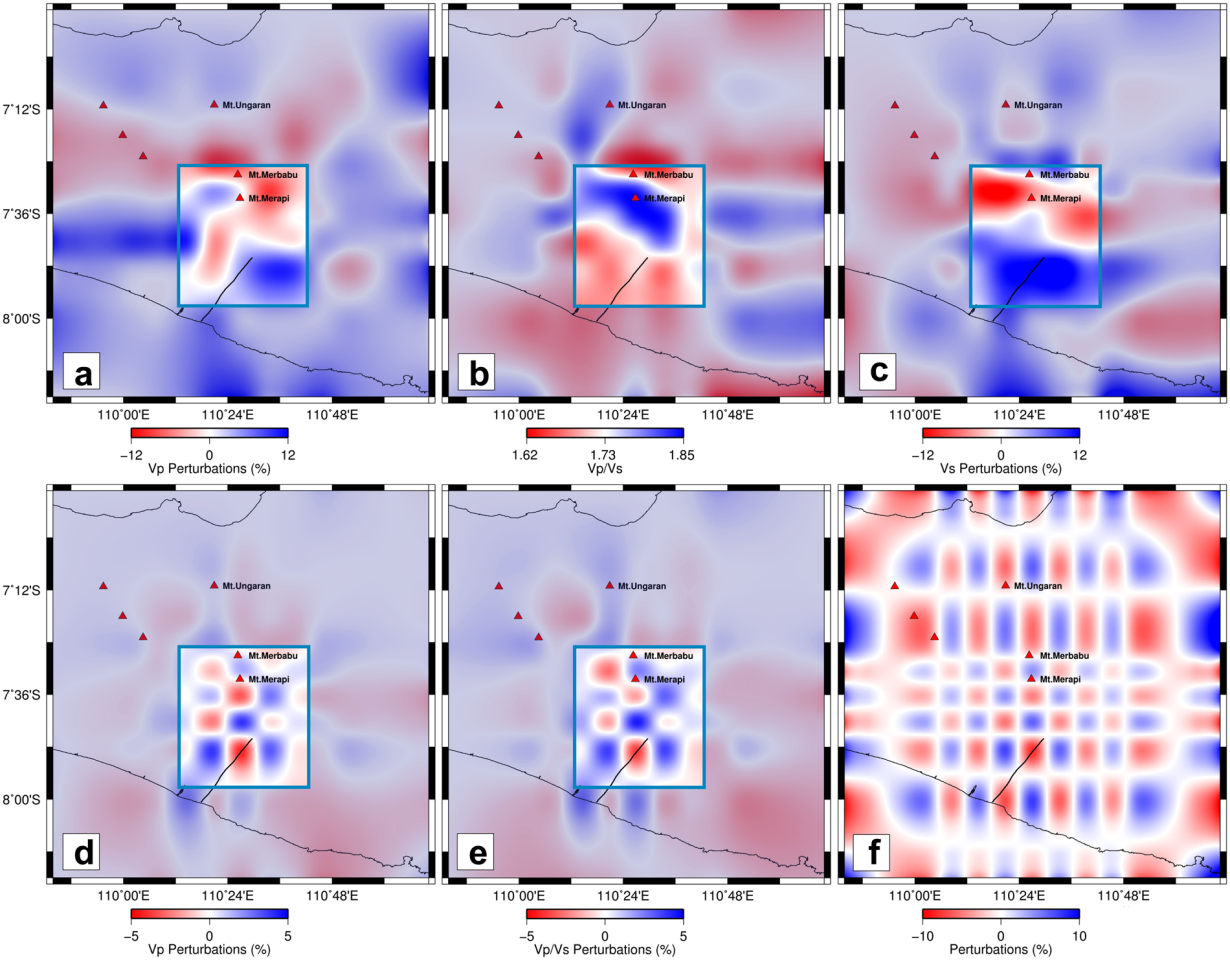
Map views of seismic velocity structure at 15 km depth below MSL. (**a**) Vp, (**b**) Vp/Vs, (**c**) Vs, (**d**) checkerboard recovery for Vp, (**e**) same as (**d**) but for Vp/Vs, and (**f**) checkerboard input model for Vp and Vp/Vs with input perturbations = +10% for both Vp and Vp/Vs. Notice that the recovery around Merapi (inside the blue box) is good owing to the DOMERAPI data; the area with poor resolution is dimmed. Here, Vp and Vs are plotted as perturbations relative to the 1D velocity model based on Koulakov *et al*.^18^ and Vp/Vs is plotted as absolute values. The high Vp/Vs is interpreted as an intermediate magma reservoir under Merapi as further illustrated in Figs 3 and 4.

**Figure 3:**
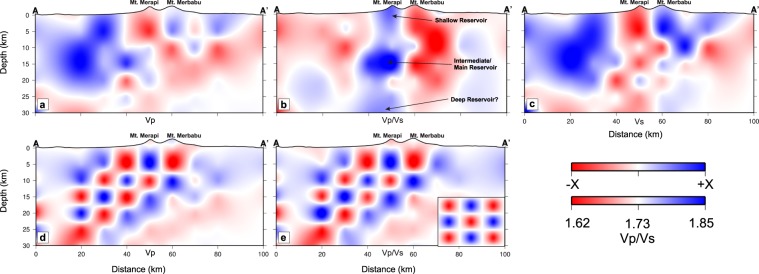
South-North vertical sections across Merapi and Merbabu. (**a**) Vp, (**b**) Vp/Vs, (**c**) Vs, (**d**) checkerboard recovery for Vp, and (**e**) same as (**d**) but for Vp/Vs. The input pattern of the checkerboard test is shown in the inset in (**e**) with input perturbations of X = 10% for both Vp and Vp/Vs as in Fig. 2. Vp and Vs are plotted as perturbations with X = 12% relative to the 1D model based on Koulakov *et al*.^18^ and X = 5% for the checkerboard recovery for Vp and Vp/Vs; while Vp/Vs is plotted as absolute values.

Our tomographic inversions reveal two pronounced anomalies directly beneath Merapi. One anomaly is located at <4 km depth where we observe low Vp, high Vp/Vs and very low Vs (Fig. 3a–c), which we term the Shallow Anomaly. A second anomaly is located at ~10–20 km depth, where we observe high Vp, very high Vp/Vs and very low Vs (Figs 2a–c and 3a–c), which we term the Intermediate Anomaly. Interestingly, the Vp/Vs tomogram suggests that another anomaly may exist near the MOHO at ≥25 km depth with low Vp, high Vp/Vs and low Vs (Fig. 3a–c), which we term the Deep Anomaly. However, we note that the resolution of this anomaly is not well constrained by our current tomographic imaging (Fig. 3d–e) due to a lack of ray sampling (see Supplementary Fig. 5).

**Figure 4:**
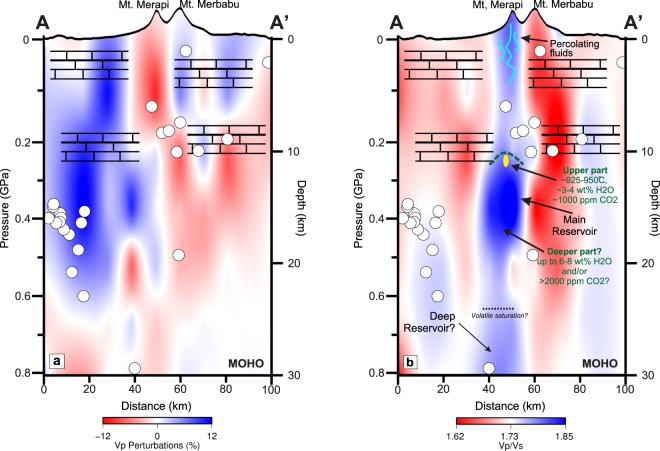
Schematic illustration of Merapi’s magma plumbing system inferred from our arrival time tomography analysis and published petrological data. (**a**) Vp and (**b**) Vp/Vs taken from Fig. 3a,b, respectively, with vertical exaggeration by a factor of 5 to emphasize vertical features. The tomogram in (**b**) shows an extensive high-Vp/Vs structure that extends from Merapi’s summit to the uppermost mantle with three main (shallow, intermediate, deep) anomalies at <4 km, at ~10–20 km, and at >25 km depth. We interpret the shallow anomaly as a fluid-rich zone, while we interpret the intermediate and deep anomalies to outline magma storage zones. We posit that the 2010 magma was sourced from the top of the intermediate reservoir (below the dashed line) at a depth just below 10 km and thus below the carbonate-dominated upper crust, with a volume of ≥1 km^3^ (corresponding to the yellow ellipse). This estimate further constrains previous estimates based on phase-equilibrium experiments^6^. Magma in this zone has a temperature of ~925–950 °C, ~3–4 wt% H_2_O, and ~1000 ppm melt CO_2_^5,6^, while magma deeper in the system may be significantly more volatile-rich and hazardous in case of ascent and eruption. We have assumed an average crustal density of 2242 kg/cm^3^ (cf.^15^) for the upper 10 km of the section, while we have estimated an average crustal density of 2900 kg/cm^3^ for the crust below (cf.^33^, for intermediate-mafic crust). Open dots depict relocated hypocenters of earthquakes recorded during the DOMERAPI experiment projected from a distance up to 20 km on both sides of the vertical section.

While relocated earthquake hypocenters at 15–25 km depth to the south of Merapi are interpreted to be related to the Opak Fault, the hypocenters at 0–10 km depth are likely to be related to volcanic activity. We note that these shallow earthquakes cluster either between the Shallow and Intermediate Anomalies, or in the low Vp/Vs anomaly to the north of our proposed Merapi magma plumbing system (Fig. 4). We speculate that these earthquakes, as well as the low Vp/Vs itself, may be related to the presence of aqueous fluids exsolved from the magmatic system that have migrated into the country rock.

Combining this new high-resolution Vp/Vs tomography with results from petrological studies leads us to propose a magma plumbing system with two main magma reservoirs that are connected by subvertical, crustal-scale fluid-rich zones (Fig. 4b). The shallow (<4 km), high Vp/Vs, low Vp anomaly within and below Merapi’s edifice could outline the presence of magma and/or fluids in intensely fractured/porous media^14,16,17^. Our seismic data cannot determine the type of liquid present, but we concur with published geophysical and petrological studies that have provided overwhelming evidence for the presence of fluids and the absence of stored magma^5,6,13,14^. Short-term ponding of magmas - i.e. for hours, days or weeks - at shallow (<3 km) depth prior to eruptions has, however, been proposed by^9,25,26^. The intermediate, high Vp/Vs anomaly concurs with several petrological studies that locate Merapi’s pre-eruptive magma reservoir in the upper- to middle crust, while the exact location of the reservoir remained highly debated^5–7,12,27,28^ (details are reported in the Supplementary Information) and the size of the reservoir unconstrained. Amphibole and clinopyroxene mineral barometry has been used to estimate the depth of Merapi’s main pre-eruptive magma reservoir^5,8,27,29^, but the reliability of these estimates has recently been called into question^6,12,30,31^ (cf. Supplementary Fig. 6). Phase-equilibrium experiments^6^ provide more robust constraints, and suggest that most magma erupted in 2010 and in other eruptions of the last ~100 years was sourced from a depth of 4–15 km (Fig. 4b). Melt inclusion hygrobarometric estimates similarly indicate intermittent magma storage depths of 6–14 km. GPS ground deformation data were used to suggest that magma erupted in 1996–1997 was sourced from a similar, but possibly shallower depth at 8.5 ± 0.5 km below and ~2 km east of Merapi’s summit^13^.

The main magma source depth (4–15 km) inferred from petrological studies thus coincides with the uppermost part of the Intermediate Anomaly at 10–20 km depth inferred from our tomography (Fig. 4b), which we interpret as a melt-rich zone that serves as Merapi’s main, pre-eruptive magma reservoir. While the size of this anomaly is close to the level of resolution of our tomographic imaging, its volume is almost certainly orders of magnitudes larger than the total volume of erupted products in 2010 (and prior eruptions)^4^, and the magma source size inferred for Merapi’s 1996–1997 eruption using GPS ground deformation data which are on the order of 1–10 × 10^6^ m^3^ (cf.^4,13^; close to the yellow ellipse in Fig. 4b). This highlights that only a small part of the magma system has been tapped during historic eruptive events including the 2010 eruption, approximately at the top of the intermediate reservoir.

The Deep Anomaly is less well-constrained in extent, but nevertheless also provides the first evidence for the location of this reservoir. The high Vp/Vs signal suggests that melt and/or fluids are present in this zone, while the weakness of the Vp anomaly may reflect poor ray path coverage. Petrological and geochemical studies had suggested that such a deep magma reservoir exists^5,6,11,12^, but previous estimates on its depth remained unconstrained^6^ or were based on untenable amphibole barometric constraints^5,11,12^ (details are shown in the Supplementary Information).

The subvertical, high Vp/Vs signal from the surface to around MOHO depth may highlight that magma storage zones are present throughout the crust as has been invoked by some studies (e.g.^8–11^). Such a distribution of magma parcels throughout the crust is possible, but most of them would have to be inactive reservoirs, as most magma erupted in 2010, but also in other eruptions of previous decades, has a crystal cargo that is texturally and compositionally strongly bimodal, indicating evacuation from one or two main zones (e.g.^5,6^). We therefore suggest instead that the crustal-scale, subvertical anomaly outlines an extensive fluid-rich zone and thus fluid fluxing in the system^6,11,29^. This interpretation is in keeping with petrological studies that have highlighted that Merapi’s system is H_2_O- and CO_2_-rich, and that deep to shallow degassing during magma ascent plays a key role in the system. If it is correct that the subvertical, high Vp/Vs anomaly outlines fluid-rich zones, it would provide unequivocal evidence that melts sourcing the system reached volatile saturation around MOHO depth, where the anomaly starts (Fig. 4b). To our knowledge, this is the first time that a fluid-fluxed zone has been seismically imaged from the mantle to the surface in great detail, i.e. showing an offset from below to above the Intermediate Anomaly and side branches above the northern edges of the intermediate and the deep reservoir, respectively. Compared with previous models based on lower-resolution seismic tomographic imaging (e.g.^18^), our model highlights that magma has a much more direct path from reservoirs at depth to the surface, which may facilitate the type of rapid ascent that led to the explosiveness of the 2010 eruption.

## Spatial constraints to reinforce forecasting and hazard assessment of future eruptions at Merapi

Unequivocal spatial and volumetric constraints on magma reservoirs throughout the crust and the connections between them is crucial for understanding the explosivity of the major eruption of Merapi on 26 October 2010 and its future hazard potential. Petrological studies^5–7^ of the 2010 eruption products all agree that its unusual explosivity was due to a much larger and much more rapid supply of magma than in previous eruptions. Our results suggest that the magma involved in the 26 October 2010 eruption evacuated the system at or near the top of the Intermediate Anomaly, while we follow others (e.g.^5,6,10^) in the suggestion that other eruptions at least within the last ~100 years were also sourced from this depth (as their eruptive products have equivalent mineral assemblages and closely comparable mineral and glass inclusion compositions), and thus that the magma erupted in 2010 had similar initial volatile contents as magmas of previous eruptions, but was less efficiently degassed in the reservoir and en route to the surface^5,7,26,29^. Our imaging, however, highlights that a large reservoir extends for a further ~10 km below historic magma source levels (Fig. 4b). A key implication of this is that a large volume of magma with a higher volatile content than that which explosively erupted in the 2010 VEI-4 event is present in Merapi’s plumbing system.

We presume that the size and the location of the main reservoir (i.e. the Intermediate Anomaly) is a long-term feature, which may be as old as or older than volcanic activity at Merapi. We highlight that we have no direct evidence or constraints for this hypothesis, but posit that pre-historic eruptions, which were commonly explosive^27^, could have been fueled by magmas from deeper levels, which should be studied in detail. Magma derived from deeper levels of the Intermediate Anomaly in the future could cause considerably more explosive and more destructive future eruptions than that from the shallowest levels if it is rapidly transported to the surface. Merapi’s basaltic andesitic magma from the top of the intermediate reservoir is moderately H_2_O- and CO_2_-rich (~3–4 wt% melt H_2_O, 1000 ppm melt CO_2_)^6,28,29^. The volatile composition of magma stored at deeper levels of the intermediate reservoir remains unconstrained, but it may be CO_2_-rich (e.g. with >2000 ppm melt CO_2_) if the magma follows an open-system degassing path (e.g. as proposed by Nadeau *et al*.^11^ and Preece *et al*.^29^) and/or H_2_O-rich (with up to ~6–8 wt% melt H_2_O) if the magmas follow a closed-system or disequilibrium degassing path (cf.^6,32^) in which case it could fuel extremely hazardous eruptions.

Our work demonstrates that high-resolution geophysical surveys are extremely powerful tools for spatially characterizing active volcanic systems such as Merapi’s, and that they are crucial in assessing hazard potential and targets for specific monitoring. Our study was carried out within the multi-disciplinary DOMERAPI project, which was designed to intimately couple geophysical and petrological insights on Merapi’s magma plumbing system; our interpretation of data shows how important this approach is for robustly characterizing such systems.

## Electronic supplementary material

Supplementary Information

## Data Availability

The DOMERAPI data used in this study are available at http://www.fdsn.org/networks/detail/YR_2013/ with citation information 10.15778/RESIF.YR2013.
