# Supplementary material for: Seismic imaging and petrology explain highly explosive eruptions of Merapi Volcano, Indonesia
Source: Sci Rep. 2018 Sep 12;8:13656. doi: 10.1038/s41598-018-31293-w (PMC6135845; doi:10.1038/s41598-018-31293-w)
Supplement: Supplementary file 1 — Supplementary Information [file 41598_2018_31293_MOESM1_ESM.pdf]

Supplementary information to the manuscript

Seismic imaging and petrology explain highly explosive eruptions of Merapi Volcano, Indonesia

By S. Widiyantoro*, M. Ramdhan, J.-P. Métaxian, P. R. Cummins, C. Martel, S. Erdmann, A. D. Nugraha, A. Budi-Santoso, A. Laurin, A. A. Fahmi

*Corresponding author, sriwid@geoph.itb.ac.id

Seismic imaging

DOMERAPI, a French-Indonesian collaborative project, deployed in the period from October 2013 to mid-April 2015 a dense seismograph network of 46 broad-band seismometers, with an inter-station distance of ~5 km, providing by far the densest coverage of seismographic stations ever used on Merapi (Fig. 1a). The DOMERAPI data combined with the data of the Indonesian Agency for Meteorology, Climatology and Geophysics (BMKG) were used simultaneously to minimize azimuthal gaps in determining event hypocenters[21].

In this study, we used a data set that consists of relocated events recorded by the DOMERAPI and BMGK stations from the same period. First, hypocenter determination of the recorded events was performed using the Geiger method[35] implemented with the HYPOELLIPSE earthquake location program[36]. In this case, the use of the BMKG data were crucial in minimizing the azimuthal gap, since the DOMERAPI stations were just placed around Mt. Merapi, while most events occurred along the Java trench to the South of the study region. The resulting events were then relocated using a double-difference earthquake location algorithm[22,37]. The jointly processed DOMERAPI and BMGK data produced a new high-quality catalog comprising 358 events used for the tomographic investigation presented here.

The following figure shows the starting 1-D P- and S-wave velocity models for tomographic inversions (Supplementary Fig. 1). An example of 3-component waveforms from an event recorded by DOMERAPI stations and picking of P- and S-wave arrival times conducted using

SeisGram2K[38] is given in Supplementary Fig. 2. Supplementary Fig. 3 shows curves depicting data variance versus model variance for selecting optimal damping values, while Supplementary Figs. 4 and 5 depict the results of synthetic tests with realistic input models and ray sampling, respectively.

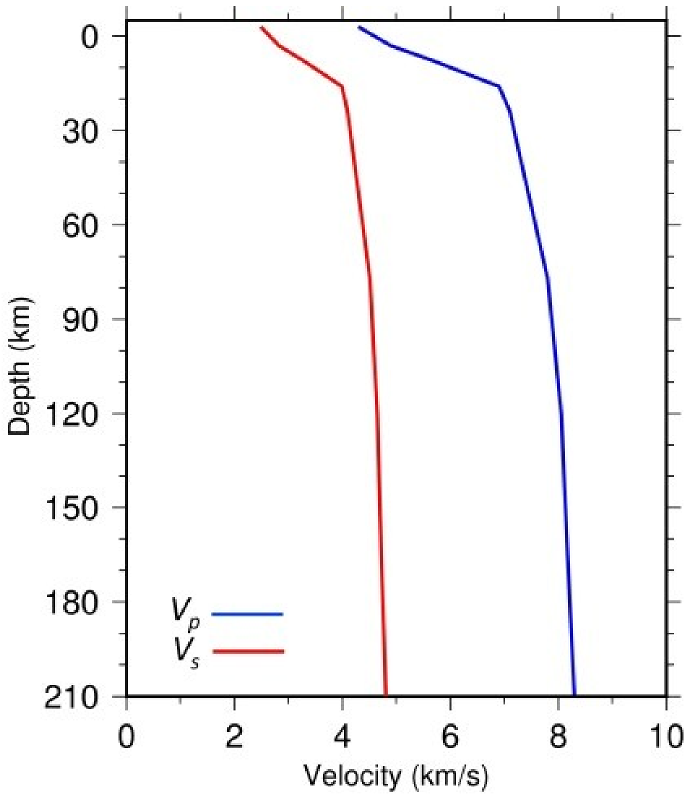

Supplementary Figure 1. *Starting 1-D P- and S-wave velocity models for tomographic inversions. The Vp model was taken from Koulakov et al.*[18] *and the associated 1-D Vs model was derived using a Vp/Vs ratio of 1.73 obtained from Ramdhan et al.*[21]

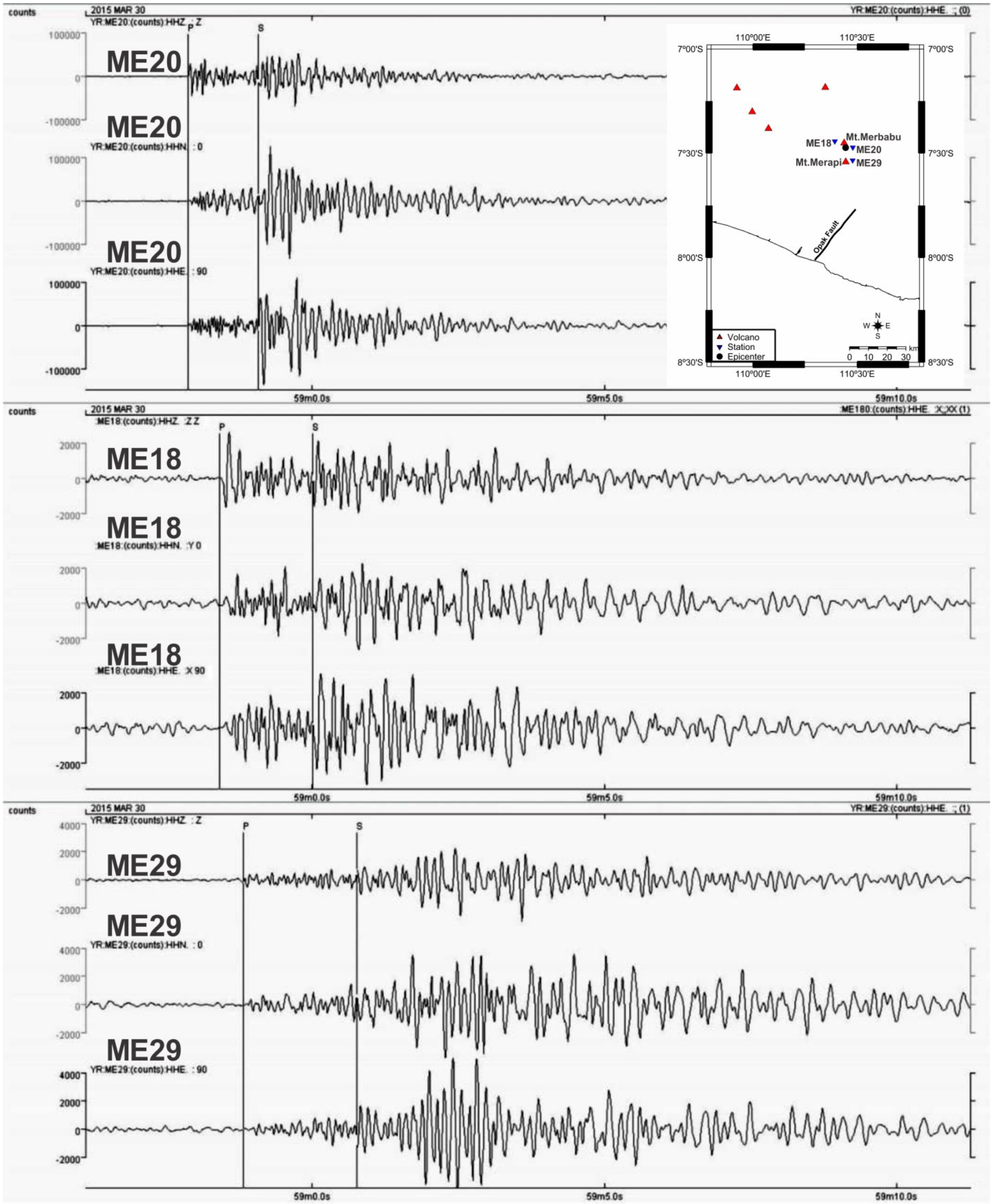

Supplementary Figure 2. *Examples of 3-component waveforms from an event recorded by 3 DOMERAPI stations and picking of P- and S-wave arrival times used in this study. Inset: locations of the event (black dot) and stations (blue reverse triangles).*

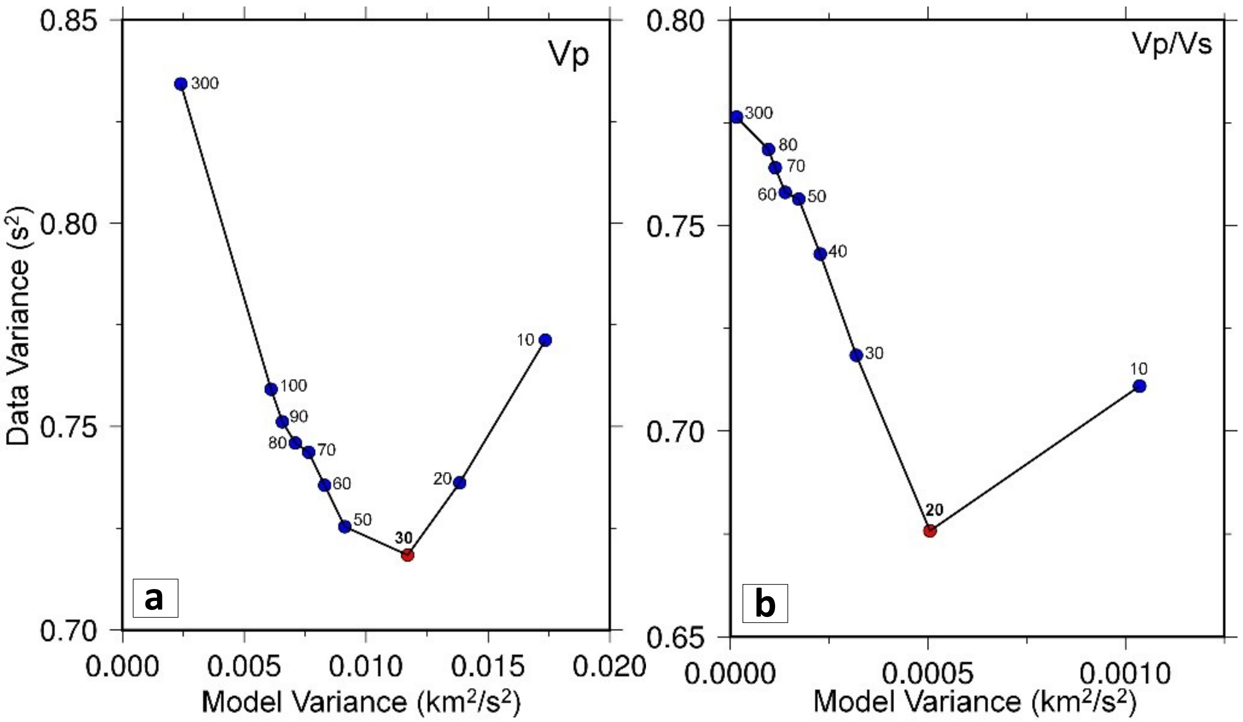

Supplementary Figure 3. *Curves showing data variance versus model variance for selecting optimal damping values. (**a**) Damping values of 30 for Vp, and (**b**) 20 for Vp/Vs (red dots), with a station damping value of 20 were selected for the inversions. The data and model variance were computed after one step iteration for indicated damping values. Note that when the damping values are too small (rightmost points), the velocity oscillates from one gridpoint to the next, and large changes in the velocity are made without corresponding large reductions in data variance*[24].

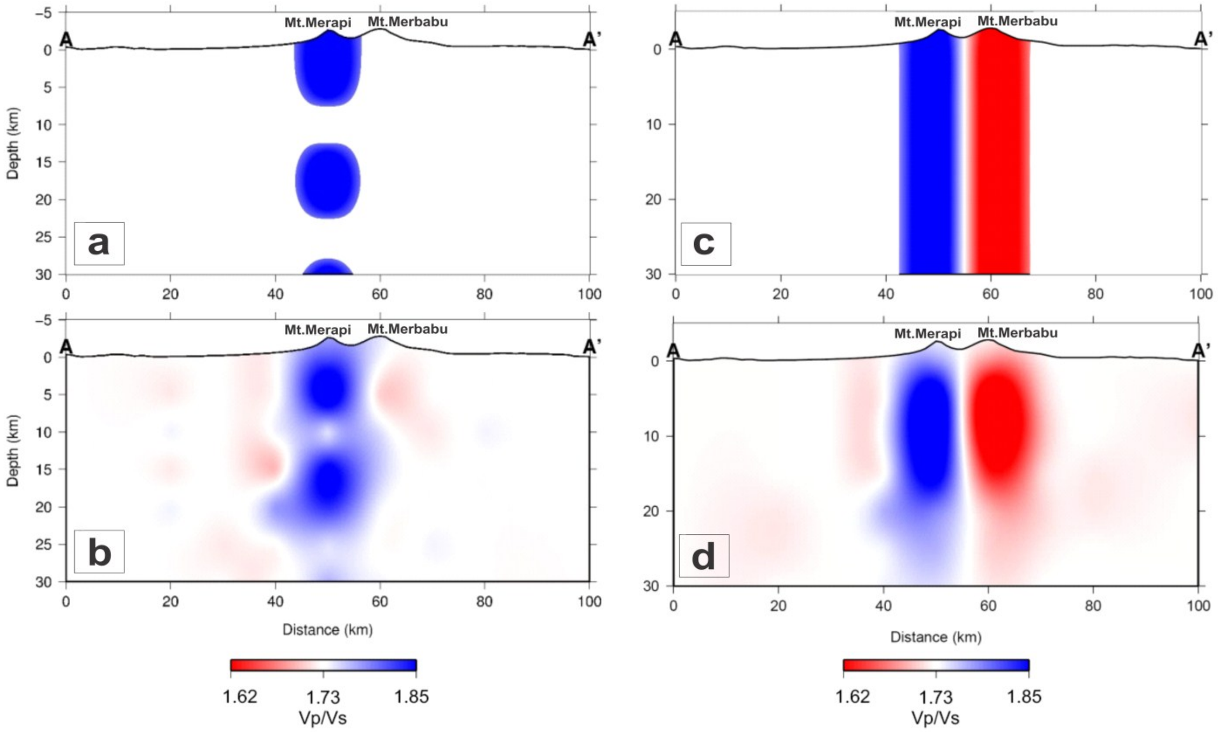

Supplementary Figure 4. *(**a**) Synthetic test with realistic input models containing representative "Shallow", "Intermediate", and "Deep" features as indicated by high Vp/Vs ratios beneath Merapi. The input perturbations are +10% relative to the reference Vp/Vs of 1.73. The Vp/Vs ratio model is plotted in absolute values. (**b**) Recovery for Vp/Vs using the same data coverage as used in the real data inversions. Notice that some smearing occurs in the vertical direction. (**c**) and (**d**) are similar to (a) and (b), but for a continuous model with lower Vp/Vs beneath Merbabu as depicted by the result of real data inversion. The input perturbations are +10% and -10% relative to the reference Vp/Vs model of 1.73. Notice that a continuous model and contrasting Vp/Vs values are well resolved, but the deep parts of the system (>~20 km) are not constrained well due to a lack of ray sampling (see Supplementary Fig. 5).*

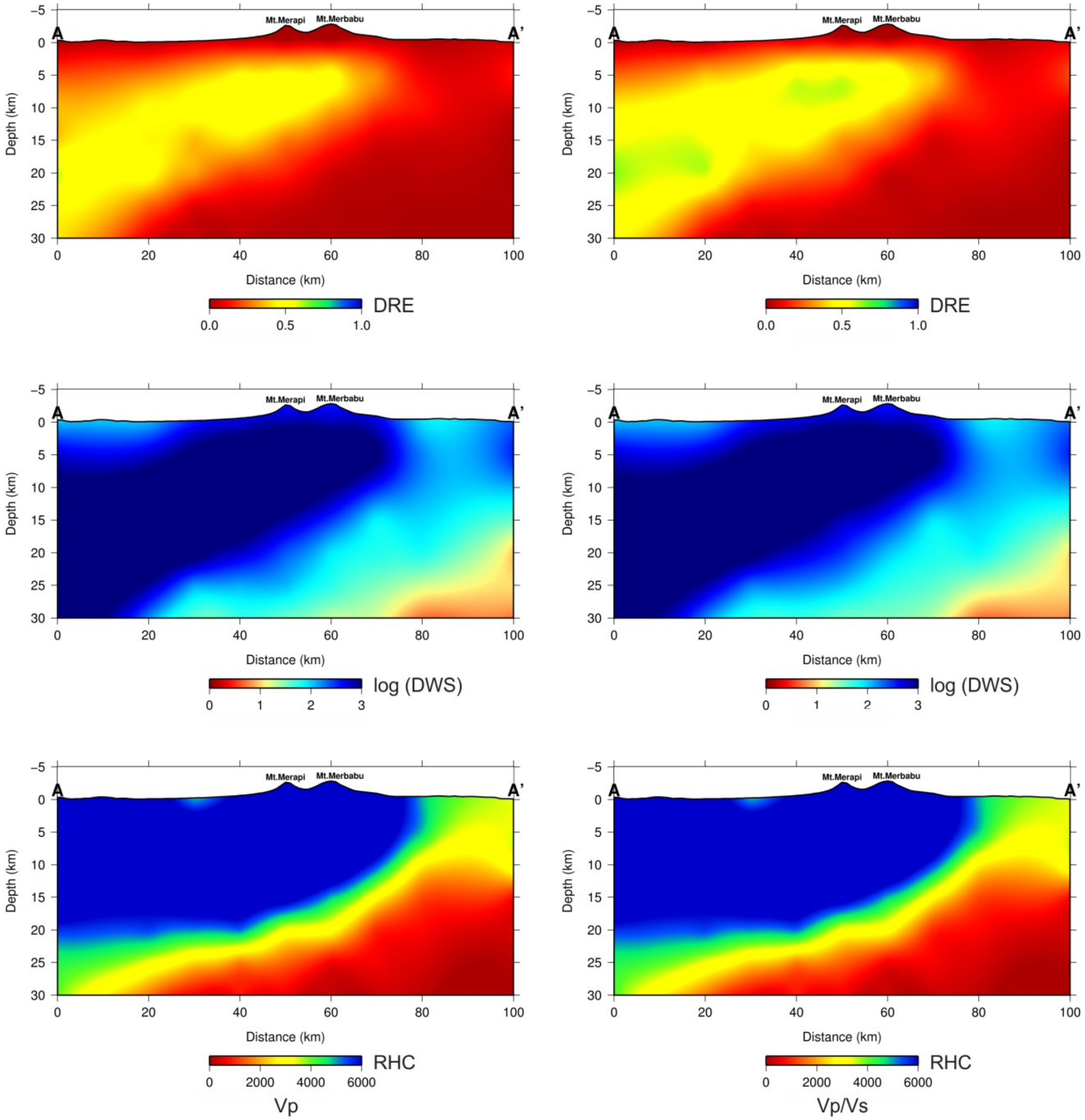

Supplementary Figure 5. *Resolution test results for Vp structure (first column) and Vp/Vs structure (second column), showing the results of diagonal resolution element (DRE, first row), derivative weight sum (DWS, second row) and ray hit count (RHC, third row). Notice that high values of DRE, DWS and RHC are an indication for high resolution of the tomographic model.*

We note that comparable examples of tomographic and interpretive advances during the past few years at large volcanoes include work at Mount Erebus[39], Yellowstone[40] and Mount St.

Helens[41]. These studies and ours are particularly noteworthy in that they are performed at some of Earth's most threating and hazardous volcanoes.

Petrology

A large number of petrological studies have aimed at characterizing Merapi's magma plumbing system in terms of magma storage pressures and depth and other intensive parameters of the system, such as melt H_2O and CO_2 contents (Supplementary Fig. 6). The key information that is ideally provided by such petrological studies is from which crustal levels magmas and/or magmatic cumulates have erupted, while they *cannot* unequivocally constrain the full spatial or volumetric extent of magma storage zones, which is crucial for future hazard assessment and mitigation. Detailed geophysical studies as those of the DOMERAPI project are unrivalled in imaging crustal-scale magma plumbing systems, while the petrological constraints are most powerful in determining from which level past and present eruptions have been sourced and how volatile-rich and thus how potentially hazardous a system is.

Models for Merapi's magma plumbing system range from those that favor storage in one or more main zones[5,6,12] to those that suggest many small magma reservoirs throughout the crust[8,9,10,11]. Studies that invoke storage in one or more zones highlight the strong bimodality of Merapi's crystal cargo and amphibole barometry, while models that posit magma storage throughout the crust are strongly influenced by calculated clinopyroxene crystallization pressures. Amphibole barometry calculates crystallization at depths of >3 km with main crystallization/storage zones at depths of ~10-15 and >23 km (A and B in Supplementary Fig. 6). The large uncertainty of the estimates (≥±500 MPa; cf.[6,31]), however, highlights that the calculated values cannot be taken as conclusive constraints (Supplementary Fig. 6). The range of amphibole composition and bimodality may equally, and indeed likely, record variation in crystallization temperature and amphibole crystallization in equilibrium with variably evolved melt instead[6]. Calculated clinopyroxene pressures have been used to infer crystallization/storage zone throughout the crust with a main zone at a depth between ~8 and 20 km (C-D in Supplementary Fig. 6). As for amphibole barometry, uncertainties are large (≥±250 MPa; cf.[6,30]) and have to be considered. We highlight moreover that the clinopyroxene barometric estimates of Chadwick et al.[8] were derived using the calibration of Nimis[42], which should not be applied to calc-alkaline systems (cf.[6,42]). The study of Chadwick *et al.*[8] also assumed that all clinopyroxene

crystallized at 1000 °C, which results in a relatively low pressure estimate (~450 MPa, i.e. a crystallization depth of ~15 km for the main, relatively evolved clinopxroxene composition, while ~700 MPa, i.e. a crystallization depth of ~25 km would be calculated for a more realistic crystallization temperature of 950 °C; cf.[6]) and which is moreover an oversimplification that inevitably results in a large spread of calculated pressures (i.e. much of the compositional variation of clinopyroxene ascribed to a pressure effect may equally record crystallization over a range of temperatures at one depth level). The clinopyroxene pressure estimates of Preece *et al.*[10] are equally based on significant temperature overestimates (i.e. they inferred crystallization at ~1025-1060 °C, which are near-liquidus temperatures for Merapi's basaltic andesites (cf.[6]), at which the bulk of clinopyroxene cannot have crystallized). Crystal-size distributions (CSDs) of clinopyroxene and plagioclase have also been used to infer crustal-wide magma storage zones and partial crystallization (F in Supplementary Fig. 6), but CSDs cannot distinguish pressure effects from variation in other intensive parameters (e.g. crystallization at variable temperature within one reservoir or crystallization in a reservoir that was only locally affected by magma recharge or fluid fluxing). Melt inclusion volatile concentrations (G and H in Supplementary Fig. 6) can be used as accurate hygrobarometers; their main limitation is that they commonly re-equilibrate during magma ascent and eruption (e.g.[43]). Maximum volatile concentrations may, but do not necessarily, highlight a zone of magma storage. Phase-equilibrium experiments have the strength that they constrain the crystallization conditions of natural system by direct comparison of mineral assemblages, phase proportions, and the composition of minerals and melts between the natural samples and experimental charges, thus using multiple lines of evidence and constraints derived for specific magma compositions (unlike constraints from classical thermobarometry; e.g.[44]). Experiments for basaltic andesite erupted at Merapi in 2010 suggest that pre-eruptive magma was sourced from pressures and depths of ≥100 and predominantly ~200 MPa, i.e. depths of ~4-11±3-4 km (I in Supplementary Fig. 6), that the magma was at ~925-950 °C and moderately volatile-rich[12], while magma from deeper levels replenished the pre-eruptive reservoir prior to eruption (e.g.[5,6,10,12]). For our interpretation of the geophysical data, we mostly rely on these experimental results, but we stress that they concur with results from GPS ground deformation data[13] and melt inclusion studies[10,11], which also have low uncertainties in estimating approximate magma storage depths.

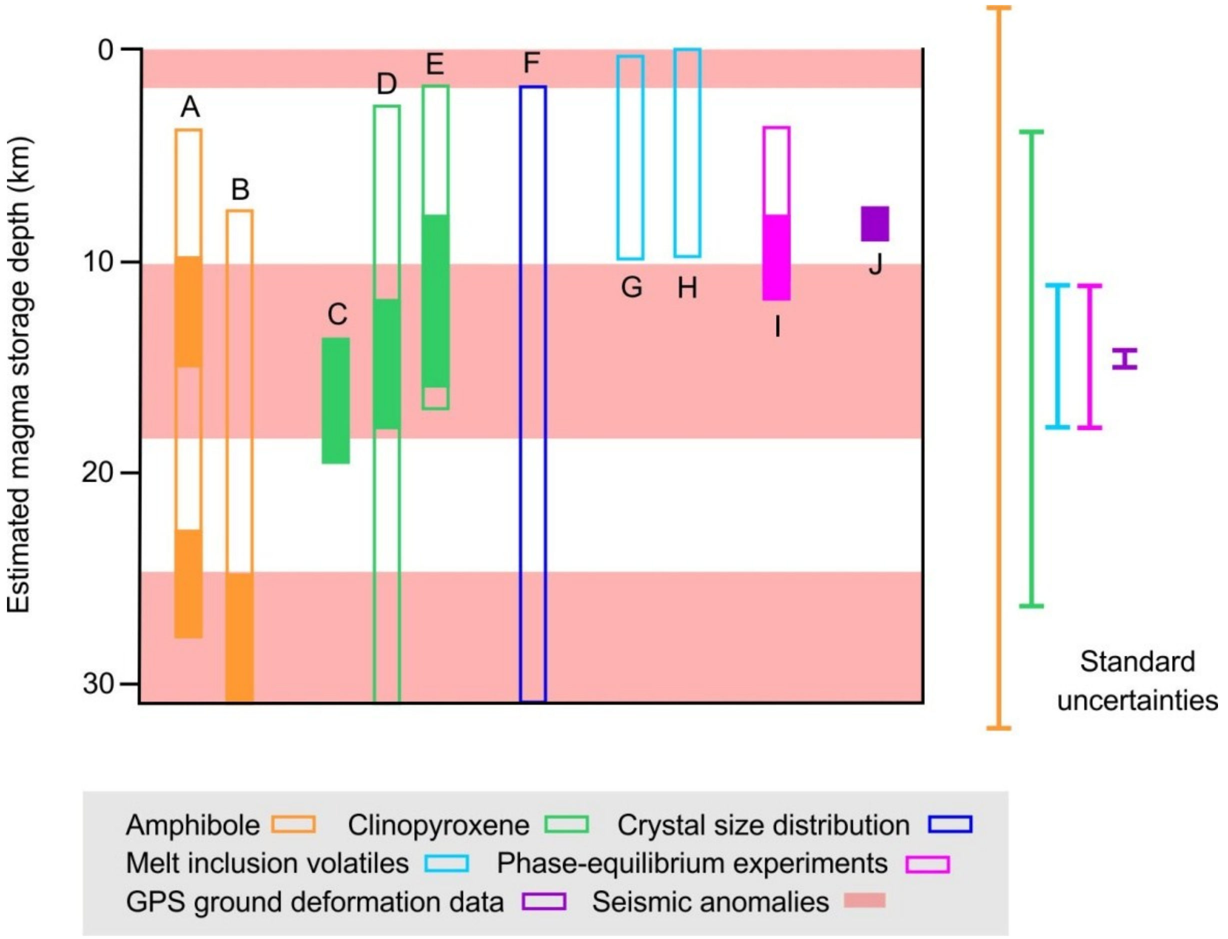

Supplementary Figure 6. *Summary of previous estimates for magma storage zones below Merapi's summit based on a range of approaches. Estimates are based on (A, B) amphibole barometry; (C-E) clinopyroxene barometry; (F) crystal size distribution and clinopyroxene barometry; (G-H) melt inclusion volatile content; (I) phase-equilibrium experiments; (J) GPS ground deformation data. Estimates are those of (A)[5], (B, H)[11], (C)[45], (D)[8], (E, G)[10], (F)[9], (I)[6], and (J)[13]. Indicated standard uncertainties are those highlighted (1) by Erdmann et al.[12] and Putirka[31] for amphibole (in orange); (2) by Putirka[30] and Erdmann et al.[6] for clinopyroxene (in green); (3) by Newman & Lowenstern[43] for melt inclusion hygrobarometry (in light blue) (4) by Erdmann et al.[6] for their phase-equilibrium experiments (in pink); and (5) by Beauducel & Cornet[13] for their GPS ground deformation data (in purple).*

Additional References

35. Geiger, L. Probability method for the determination of earthquake epicenters from the arrival time only (translated from Geiger's 1910 German article). *Bull. St. Louis Univ.* **8**, no. 1, 56–71 (1912).

36. Lahr, J. C. HYPOELLIPSE: a computer program for determining local earthquake hypocentral parameters, magnitude, and first-motion pattern (Y2K compliant version). *U.S. Geol. Surv. Open-File Rep.* **99–23**, 123 pp (1999).

37. Waldhauser, F. *hypoDD* -- A computer program to compute double-difference hypocenter locations. *U.S. Geol. Surv. Open File Rep*. **01–113**, 1–25 (2001).

38. Lomax, A. & Michelini, A., Mwpd: A duration-amplitude procedure for rapid determination of earthquake magnitude and tsunamigenic potential from P waveforms. *Geophys. J. Int*., **176**, 200–214, doi:10.1111/j.1365-246X.2008.03974.x (2009).

39. Zandomeneghi, D. *et al*. Internal structure of Erebus volcano, Antarctica imaged by high-resolution active-source seismic tomography and coda interferometry. *J. Geophys. Res.* **118**, 1067–1078, doi:10.1002/jgrb.50073 (2013).

40. Huang, H. *et al*. The Yellowstone magmatic system from the mantle plume to the upper crust. *Science* **348(6236)**, 773–775, doi:10.1126/science.aaa5648 (2015).

41. Kiser, E. *et al*. Magma reservoirs from the upper crust to the Moho inferred from high-resolution Vp and Vs models beneath Mount St. Helens, Washington State, USA. *Geology* **44(6)**, 411–414, doi:https://doi.org/10.1130/G37591.1 (2016).

42. Nimis, P. Clinopyroxene geobarometry of magmatic rocks. Part 2. Structural geobarometers for basic to acid, tholeiitic and mildly alkaline magmatic systems. *Contrib. Mineral. Petrol.* **135**, 62–74, DOI https://doi.org/10.1007/s004100050498 (1999).

43. Newman, S. & Lowenstern, J. B. VOLATILECALC: a silicate melt-H_2O–CO_2 solution model written in Visual Basic for excel. *Comp. Geosci.* **28**, 597–604, https://doi.org/10.1016/S0098-3004(01)00081-4 (2002).

44. Blundy, J. & Cashman, K. Petrologic reconstruction of magmatic system variables and processes. *Rev. Mineral. Geochem.* **69**, 179-239, DOI: 10.2138/rmg.2008.69.6 (2008).

45. Gertisser, R. Gunung Merapi (Java, Indonesien): Eruptionsgeschichte und Magmatische Evolution eines Hochrisiko-Vulkans. Ph.D. Thesis, Universität Freiburg, Germany, 382 pp. (2001).
